# A novel mutation site in STAT in a chronic mucocutaneous candidiasis pediatric patient with disseminated cryptococcosis: Case report and review of the literature

**DOI:** 10.1002/pdi3.58

**Published:** 2024-05-14

**Authors:** Chuan Gan, Yu Nie, Gaihuan Zheng

**Affiliations:** ^1^ The Infection Department of Children's Hospital of Chongqing Medical University, National Clinical Research Center for Child Health and Disorders Ministry of Education Key Laboratory of Child Development The First Batch of Key Disciplines on Public Health in Chongqing Chongqing China

## 1 INTRODUCTION

Chronic Mucocutaneous Candidiasis (CMC) is a complex clinical syndrome with the skin, mucous membrane, and fingernails frequently infected with fungi, especially candida. The patients often have endocrine or immune disorders. Almost all CMC patients are related to genetic or acquired T cell dysfunction. *STAT1* gene mutation is an important cause of CMC, and more than 50 mutation sites have been reported.^1^ Fungal infection in CMC patients is mainly manifested as superficial cutaneous mucocutaneous candidiasis. Deep fungal infection can occur in some CMC patients, such as invasive candidiasis, histoplasmosis, and Pneumocystis jiroveci infection, while it is very rare for CMC patients to have invasive cryptococcal infection at the same time. We report that this CMC pediatric patient with a new *STAT1* gene mutation site had superficial cutaneous fungal infection, as well as intracranial and hematogenous cryptococcal infection.

## 2 MATERIALS AND METHODS

### 2.1 Medical history data

The patient was a child, boy, aged 5 years 8 months. He was admitted to the department of infection of Children's Hospital of Chongqing Medical University with a complaint of “fever for 15 days, neck pain on the left side for 13 days, and abdominal pain for 1 week”. The patient manifested as persistent moderate to high fever. Neck pain was on the left side with local swelling, tenderness, no redness, and ulceration. Abdominal pain was in total abdomen, paroxysmal aggravation, obvious tenderness, and muscle tension. No effect with ceftriaxone sodium used for 5 days and amoxicillin clavulanate potassium for 4 days before admission. The patient was G1P1, full term, and birth weight 3.1 kg. His mother was healthy and lived in a newly decorated house for half a year during her pregnancy. The patient suffered from thrush at 7 months, although many times of topical treatment with nystatin, it had not recovered. He who suffered from onychomycosis on both toenails and the fourth toe of his left foot had repeated redness, swelling, and pain in recent half a year. The patient had no family history of genetic disease.

On physical examination, normal growth and development, medium nutrition, normal mental reaction, an enlarged lymph node with tenderness and about 2 × 3 cm in size was observed on the left side of the neck, thrush could be seen in the oral mucosa, slightly distended abdomen, tenderness and muscle tension in the whole abdomen, and the fourth toe of the left foot was red and swollen with tenderness.

The ethical approval of the case report had been approved by the medical ethics committee of Children’s Hospital of Chongqing Medical University. At the same time, the informed consent of the patient's family had been obtained.

### 2.2 Laboratory and auxiliary examinations

#### 2.2.1 Blood biochemical tests

We gave the patient routine biochemical tests. The renal function, electrolyte, blood lipid, blood glucose, hemagglutination, T3, T4, TSH, alpha fetoprotein, gonadotropin, cortisol, and insulin were normal. The results of blood routine examination at the beginning of admission showed that the proportion of peripheral blood lymphocytes was decreased. The abnormal results of blood biochemical tests at the beginning of hospitalization are shown in Table 1.

**TABLE 1:** The abnormal results of blood biochemical tests at the beginning of hospitalization.

Tested items	Results	Normal reference value
Peripheral blood leukocyte cerebrospinal fluid biochemistry	12.230 × 10^9^/L	4.000∼10.000 × 10^9^/L
Percentage of peripheral blood lymphocytes	20%	30%–60%
Hemoglobin in peripheral blood	78.000 g/L	110.000∼160.000 g/L
Peripheral erythrocytes	3.120 × 10^12^/L	3.800–5.500× 10^12^/L
C‐reactive protein	18.000 mg/L	<8.000 mg/L
Procalcitonin	1.484 ng/mL	<0.050 ng/mL
Erythrocyte sedimentation rate	84.000 mm/1 h	0.000∼15.000 mm/1 h
Serum ferritin	1283.000 ng/mL	28.000∼365.000 ng/mL
Alanine aminotransferase	112.200 U/L	0–50.000 U/L
Parathyroid hormone	<3.000 pg/mL	10.000–69.000 pg/mL
Adrenocorticotropic hormone	18.900 pg/mL	<46.000 pg/mL
Antithyroid globulin antibody	389.800 IU/mL	<18.100 IU/mL
Thyroid peroxidase antibody	121.7I0 U/mL	<7.000 IU/mL

#### 2.2.2 Etiology tests

**FIGURE 1:**
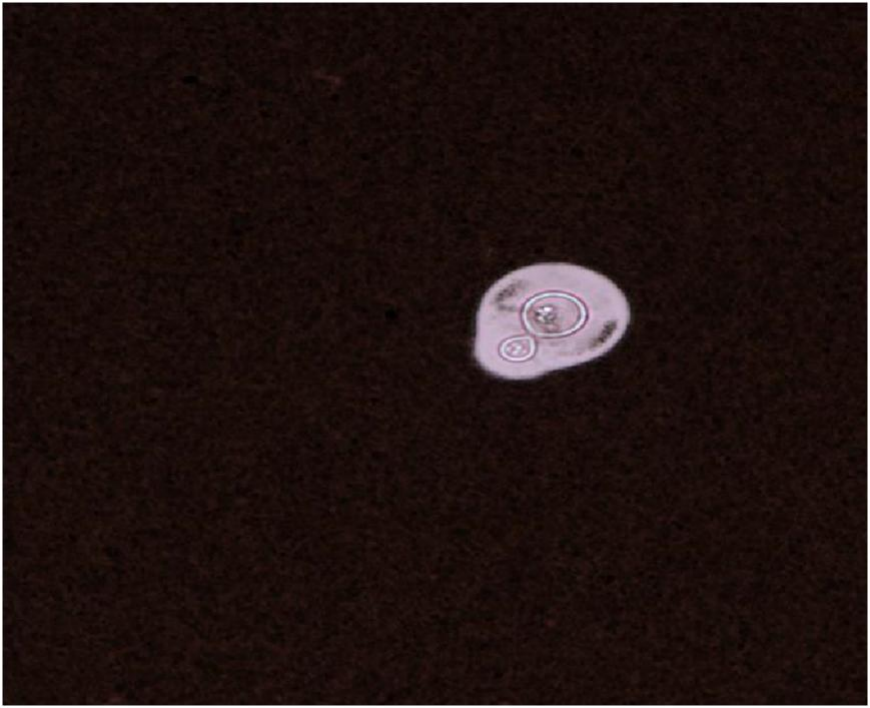
Cryptococcus neoformans was found in blood culture and CSF smear(×400).

We gave the patient etiology related tests. Some of the patient's etiological tests included T‐SPOT‐TB, blood fungus 1‐3 *β*‐D‐glucan, virus antibody No. 4 + EBV, hepatitis markers + HIV + syphilis, respiratory syncytial virus, adenovirus, influenza B, parainfluenza antigen, Mycoplasma pneumoniae, Chlamydia pneumoniae PCR, sputum TB‐PCR, and sputum culture were all negative. Sputum influenza A (++) Cryptococcus neoformans was found in the patient's blood by blood culture. Cryptococcus neoformans was also found in the cerebrospinal fluid (CSF) of the patients Figure 1.

#### 2.2.3 Preliminary detection of immune function

We did a preliminary examination of the child patient's immune function, and the blood autoantibody test result was negative. There was no obvious abnormality in lymphocyte classification. Our test results showed that there was no abnormality in the classification of his venous blood lymphocytes. The detection result of phagocytic function of leukocytes (nitroblue tetrazolium test) was also normal. The cytokine test results show that IL‐17a is zero. Tables 2, 3, 4.

**TABLE 2:** The child patient's blood lymphocyte classification test result.

Tested items	Results	Normal reference value
CD3+	78.37%	55.00∼78.00%
CD3+CD8+	38.94%	19.00∼34.00%
CD3+CD4+	33.39%	27.00∼53.00%
NK	3.70%	4.00∼26.00%
CD19+	15.89%	10.00∼31.00%
CD4/CD8	0.86	0.98–1.94

**TABLE 3:** The child patient's levels of immunoglobulin and complements.

Tested items	Results	Normal reference value
IgG	8.01 g/L	5.28–21.9 (g/L)
IgA	1.10 g/L	0.61–3.45 (g/L)
IgM	1.23 g/L	0.48–2.26 (g/L)
IgE	2.00 IU/ML	<150 (IU/ML)
C3	1.28 g/L	0.7–2.06 (g/L)
C4	0.27 g/L	0.11–0.61 (g/L)

**TABLE 4:** The result of nitroblue tetrazolium test of the child patient.

Name	Result (%)
No stimulation in normal people	22
No stimulation in the patient	19
LPS stimulation in normal people	33
LPS stimulation in the patient	29

#### 2.2.4 B‐ultrasonography, imaging, and pathological examinations

We performed the necessary B‐ultrasonography, imaging, and pathological examinations on the patient. The results of abdominal B‐ultrasonography showed intraabdominal lymphadenopathy, accumulation of omentum in midabdomen, and a small amount of abdominal dropsy. The B‐ultrasonic examination of the neck showed lymphadenopathy on the left side of the neck and a slightly enlarged right cervical lymph node. There was no abnormality in the bone radiographs of the head and limbs.

**FIGURE 2:**
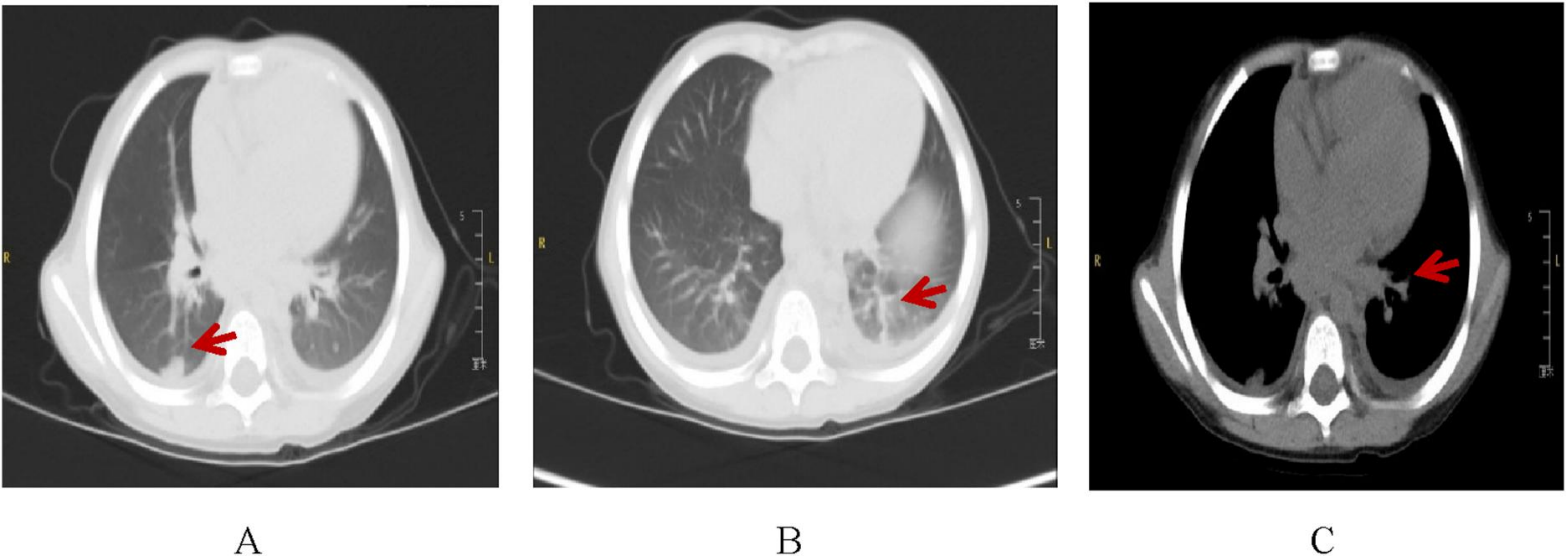
The results of enhanced CT of the child patient's chest. The arrows A&B refer to the inflammatory focus of the lung. The arrow of C refers to the enlarged lymph nodes.

The results of enhanced CT of chest showed inferior lobar lesions of bilateral lungs with segmental pulmonary consolidation and atelectasis, bilateral pleural effusion, and multiple lymphadenopathy in bilateral supraclavicular fossa, mediastinum, hilum, and around thoracic aorta. Figure 2.

**FIGURE 3:**
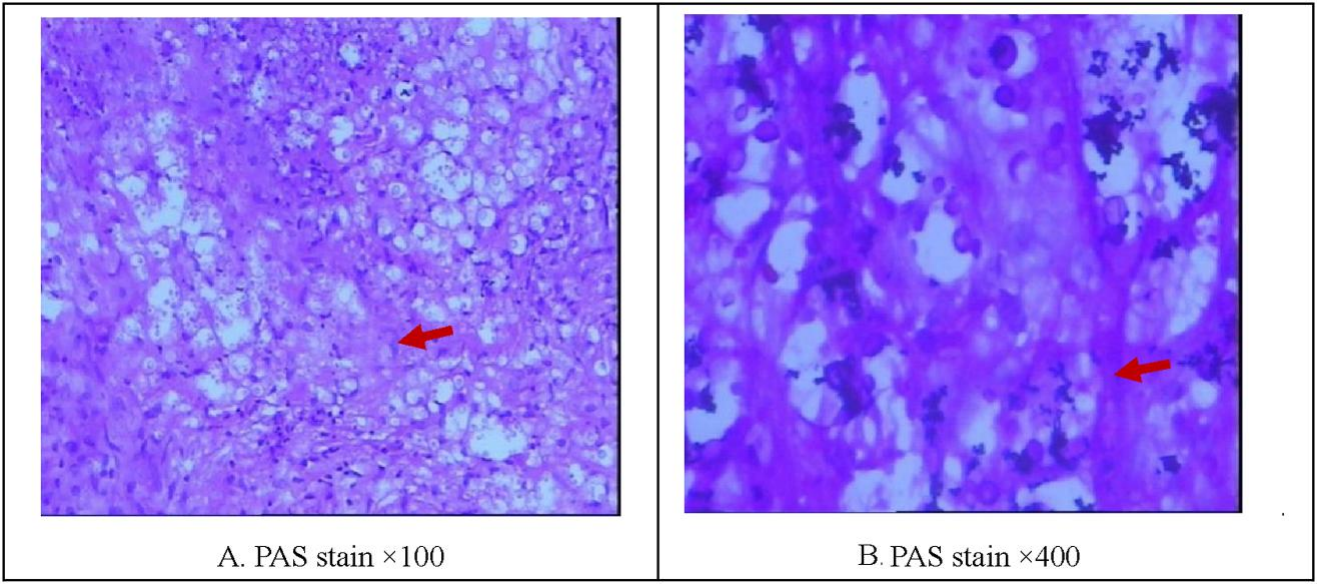
Pathological findings of the patient's cervical lymph nodes. Destruction of lymphoid structure, massive histiocytosis, and granulomatous structure. Large necrosis and inflammatory exudation can be seen. A large number of round transparent bodies can be seen in the histoplasm. Immunohistochemistry: CD68Histiocytic cells(+), PAS(+).

In order to exclude tumor diseases, the lymph nodes of the patient's neck were surgically excised for pathological examination. The pathological examination results showed that the cervical lymph node pathology was consistent with cryptococcus infection. Figure 3.

#### 2.2.5 Gene detection

We did further genetic tests on the child patient. With the consent of the patient's parents, the peripheral blood samples of the patient and his parents were sent to Beijing Mejino Gene Testing Company for gene detection. Second generation gene sequencing was performed on the patient himself. The peripheral blood of the parents was used for first‐generation gene sequencing to verify. Average sequencing depth on target of gene sequencing was 379.68, and coverage of target region of gene sequencing was 99.85%. The partial gene sequence of *STAT1* exon in this patient is as follows:

TGAAGCCGGAGTGTGTACATATATGTATATCAGTTACATTTCTACTATGAATATATTAATAAAAGACTCTCAGATATTCTCAGTAAGAGTTCATATTAAAGTGCCCCCTGAAAAATTATTTCCTCAAAAGCACCCTATATAACAGTTTTTATAAAAGGAAACTAGGGGTACAAACTACGTGACAGGTGATGTATGGGATGCCATCTTTCCCTTGTTACCTCAACTTCACAGTGAACTGGACCCCTGTCTTCAAGACCAGCGGCCTCTTAGGGTGCGTTGGCATGCAGGGCTGTCTTTCCACCACAAACGAGCTGCAAATACCCAGCAAAGGATAGATAAGTTAGCATTTCCATTAAGGTTGAGGCAATCACAATGATTTTCCTGAAAGAAAAAAGGGGGTTAGAGAGTACGATCTAAAAAATCATTTGAAAGATTCTGACAAGTTGGATGAAACAAAGTGA.

**FIGURE 4:**
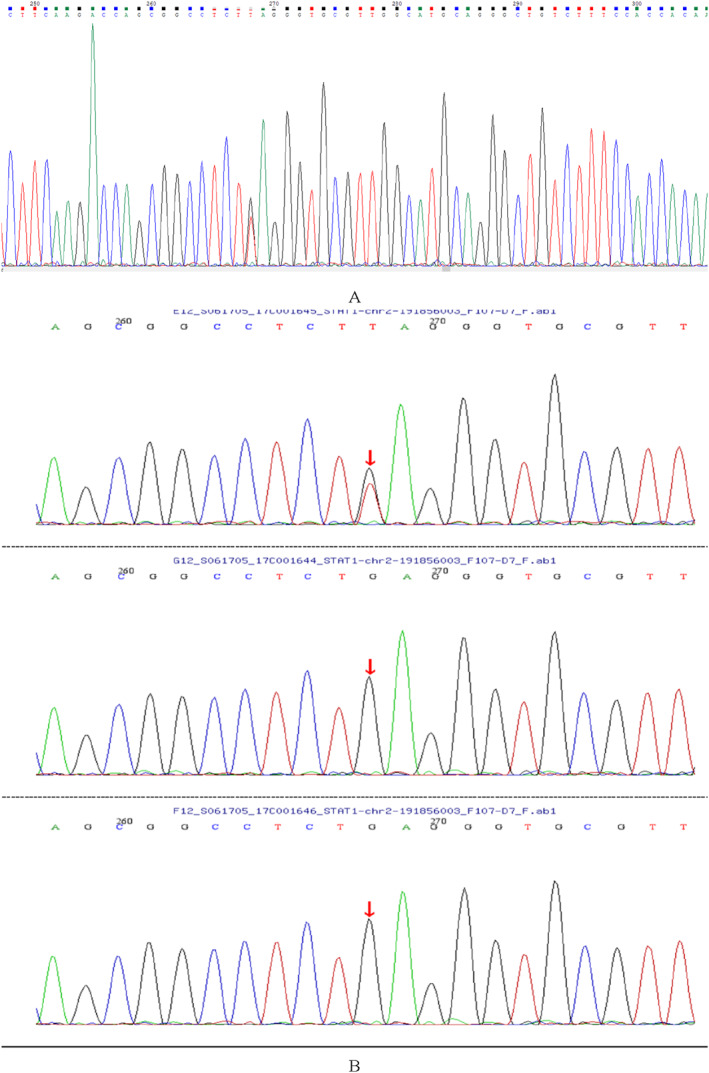
Sequencing results of *STAT1* gene mutations. A: Partial sequencing results of *STAT1* exon. B: Heterozygous mutation C. 988C > A exists in the child patient; Parents have no C. 988C > A mutation.

The results showed that there was a heterozygous mutation in *STAT1* gene: C. 988C > A (nucleotide 988 in coding region changed from cytosine to adenine), resulting in the change of amino acid P. Q330 K (amino acid 330 changed from glutamine to lysine), which was a missense mutation. Through comparative analysis in the single nucleotide polymorphism database of National Center for Biotechnology Information, it was found that there was no information on this mutation site and no such mutation in the gene recombination of thousands of people, which indicate that the mutation does not belong to polymorphic loci and occurs in a very low frequency in the population. This mutation site has not been reported in literature, and it is a new mutation site of *STAT1* gene. The gene sites of the patient's parents are normal. Figure 4.

## 3 RESULTS

After hospitalization, the child was diagnosed with disseminated cryptococcosis (cryptococcal septicemia, cryptococcal encephalitis, cryptococal pneumonia, and cryptococcal peritonitis), cutaneous and mucosal candidiasis, and hypoparathyroidism. After targeted and standardized antifungal treatment, the abnormal blood biochemical test results of the patient returned to normal. The prognosis of children was good. The fungal infection did not recur after the 3‐year follow‐up.

## 4 DISCUSSION

CMC is a group of immunodeficiency diseases related to T cell dysfunction.^2^ CMC patients are more likely to be infected with bacteria, virus and fungus, especially candida albicans. In children, the pathogenesis of CMC is mainly related to the dysfunction of C‐type lectin receptor(dectin) and IL‐17 pathway by congenital genetic defects. Clinical presentation, including the presence of other infections, helps distinguish the primary cause of CMC, and molecular diagnosis is necessary for confirmation.^3^

The defense of pathogen infection include macrophages, cytotoxic lymphocytes, and natural killer cells, especially macrophages. Macrophages are critical for controlling intracellular microorganisms, including mycobacteria and dimorphic fungi, by mediating antigen presentation, T‐lymphocyte activation, cytokine production, and intracellular killing.^4^ Pathogen engulfment by macrophages results in the production of IL‐12p70, which stimulates T cells and NK cells through its receptor IL‐12R to secrete interferon (IFN)‐γ. Then, IFN‐γ acts through its receptor to activate *STAT1*, which translocates to the nucleus and upregulates the transcription of IFN‐γ‐related genes.^5^

The *STAT1* mutant alleles were gain‐of‐function by impairing nuclear dephosphorylation of activated *STAT1* and led to increased *STAT1*‐dependent cellular responses to IL‐27, IFN‐γ, and IFN‐α/β, which potently inhibit Th17 development. As a result, patients with *STAT1* mutations had low proportions of circulating IL‐17+ and IL‐22+ T cells, and their peripheral blood mononuclear cells secreted very low amounts of IL‐17A, IL‐17 F, and IL‐22 upon stimulation.^6^ In addition, IL‐17 and IL‐22 can stimulate the production of chemokines, defensins, and inflammatory cells, thus playing a defensive role against candida. Th17 cells play an indispensable protective role in CMC.^7^, ^8^ *STAT3* triggered the differentiation of Th17 cells and induced the production of related cytokines, such as IL‐17, IL‐6, and IL‐21, which mediates protective immunity against fungi. *STAT1* gain of function mutations affecting the *STAT3*/interleukin 17 (IL‐17) pathway causes selective susceptibility to fungal (candida) infections. Mutations affecting the *STAT3*/interleukin 17 (IL‐17) pathway cause selective susceptibility to fungal (candida) infections.

CMC patients were maily characterized by superficial fungal infections, with 95% being candida albicans. Invasive fungal infections were very rare.^9^ In this case, due to recurrent skin, mucosa, and nail fungal infections since 7 months old, we suspect that the patient may have primary immunodeficiency. Therefore, we did a preliminary screening of the immune function for the child patient. But preliminary screening did not find any abnormalities. There was no obvious abnormality in lymphocyte classification. Normal levels of immunoglobulin and complements were observed. The detection result of phagocytic function of leukocytes (nitroblue tetrazolium test) was also normal. We did further gene tests. Gene tests showed that there was a heterozygous mutation in *STAT1* gene: C. 988C > A. Due to the children patient's repeated superficial fungal infections, as well as autoimmune and endocrine diseases, we considered the *STAT1* mutation as the pathogenic mutation, which was the reason for the patient's CMC related manifestations. Immune function screening plays an important role in the diagnosis of immunodeficiency disease. However, in many CMC patients, initial immune function screenings were normal, such as lymphocyte classification (T, B, and NK cells) and immunoglobulin classification. Only some patients had abnormal lymphocyte classification and immunoglobulin levels.^10^ It suggests that detecting of immunoglobulin and lymphocyte classfication levels in patients is not decisive for the existence of CMC. It is necessary to detect the related gene if clinical tests indicate CMC.

CMC starts during infancy for 60%–80% of the patients, whereas late onset is rare.^11^ The patient had recurrent superficial fungi infection of skin, nails, and mucous membranes since 7 months of life. CMC was not considered after diagnosis and treatment in many hospitals with the local treatment. It is suggested that attention should be paid to similar patients. This patient was accompanied by autoimmune or endocrine disorders, such as increased antithyroglobulin antibody and antithyroid peroxidase antibody, decreased adrenocorticotropic hormone and parathyroid hormone, suggesting thyroid autoimmune response, parathyroid disease, and adrenal cortical disease. These related immune reactions or diseases have also been reported in CMC in the past.

The patient's blood analysis showed that there were no obvious abnormalities in blood cells and immune cytopenia. However, the percentage of lymphocytes in leukocyte classification was lower (11%–24%) and the lymphocyte count decreased. In previous studies, T lymphocyte and B lymphocyte decreased in CMC patients with *STAT1* functional acquired mutation. The study found that only some patients with CMC had lymphocytopenia, and 70%–80% of patients with CMC had a normal lymphocyte count and B lymphocyte function. Some abnormal blood biochemical tests result in the early stage of hospitalization, such as the increase of CRP, transaminase, and ferritin, and the decrease of hemoglobin could be related to the status of fungal infection. After the cryptococcal infection was cleared, these test indexes returned to normal.

CMC patients more rarely can develop invasive fungal infection, only a few literature reports have been made in previous studies, for example, disseminated coccidioidomycosis, histoplasmosis,^10^, ^12^ or disseminated mucormycosis.^13^ *STAT1* gain of function CMC complicated with cryptococcal infection is rarely reported. There were only a few related reports in literature.^12^ The patient found that there were no fungal susceptibility factors, such as long‐term repeated use of antibiotics, long‐term use of glucocorticoids, immunosuppressants, and HIV infection. Cryptococcus was found in cerebrospinal fluid smear and blood culture after admission, and cryptococcus infection was confirmed. However, patients with negative G test suggest that G test negative for CMC cannot be excluded from invasive fungal infection.

This patient's central nervous system cryptococcal infection was cured after a combination of amphotericin‐B and fluorocytosine for 8 weeks of intensive treatment,followed by fluconazole consolidation treatment for 1 year and 4 months. For CMC patients, in addition to antifungal treatment for their fungal infection complications, some CMC patients can also receive immunotherapy by using Granulocyte colony‐stimulating factor(G‐CSF) or other cytokines. Except for complications, there are hematopoietic stem‐cell transplantation、G‐CSF、and cytokines immunotherapy method.^14^, ^15^, ^16^, ^17^ At present, there are few clinical patient‐based studies and few data from large‐scale multicenter studies, and there is still some controversy about the clinical efficacy. Therefore, it needs to be further explored in future research.

## AUTHOR CONTRIBUTIONS

Gaihuan Zheng and Chuan Gan traced the case, and were responsible for identifying the materials, Yu Nie participated in writting the manuscript and analyzing the sequence. All authors read and approved the final manuscript.

## CONFLICT OF INTEREST STATEMENT

The authors declare that they have no competing interests.

## ETHICS STATEMENT

The patient provided written informed consent for the publication of the case report and accompanying images. (2024) Ethical review (Research) (117).

## CONSENT FOR PUBLICATION

Written informed consent for publication of the clinical details including the medical history, bacteria cultures, pictures, videos and text was obtained from his parents. The parents gave their written consent for their child’s personal and clinical details along with any identifying images to be published in this study.

## Data Availability

The data that support the findings of this study are available from the corresponding author upon reasonable request.
